# The role of artificial intelligence in advancing population-based cancer registration

**DOI:** 10.1016/j.scib.2026.02.012

**Published:** 2026-03-30

**Authors:** Shuai Ding, Mingyuan Liu, Hao Wang, Cheng Song, Luyue Zhao, Zhihao Yang, Yue Wang, Yifan Wang, Haitao Cui, Zihao Liu, Dongrun Liu, Tomohiro Matsuda, Megumi Hori, Dimitris Katsimpokis, Gijs Geleijnse, Xavier Farré, David S Morrison, Yaogang Wang, Siwei Zhang, Meicen Liu, Qiushuo Geng, Ling Ni, Kexin Sun, Bingfeng Han, Shaoming Wang, Ru Chen, Li Li, Hiromi Sugiyama, Kyu-Won Jung, Yongyue Wei, Wanqing Chen, Isabelle Soerjomataram, Freddie Bray, Hongmei Zeng, Jie He

**Affiliations:** a School of Management, Hefei University of Technology, Hefei 230009, China; b National Cancer Center/National Clinical Research Center for Cancer/Cancer Hospital, Chinese Academy of Medical Sciences and Peking Union Medical College, Beijing 100021, China; c Engineering Research Center for Intelligent Decision-Making & Information System Technologies, Ministry of Education, Hefei 230009, China; d Key Laboratory of Process Optimization and Intelligent Decision-Making, Ministry of Education, Hefei 230009, China; e Philosophy and Social Sciences Laboratory of Data Science and Smart Society Governance, Ministry of Education, Hefei 230009, China; f Center for Cancer Registries, Institute for Cancer Control, National Cancer Center, Tokyo 104-0045, Japan; g Department of Research and Development, Netherlands Comprehensive Cancer Organisation, Utrecht 3511 CV, Netherlands; h Public Health Agency of Catalonia, Lleida 25006, Spain; i Public Health Scotland, Edinburgh EH12 9EB, UK; j School of Public Health, Tianjin Medical University, Tianjin 300070, China; k School of Integrative Medicine, Public Health Science and Engineering College, Tianjin University of Traditional Chinese Medicine, Tianjin 301617, China; l National Institute of Health Data Science at Peking University, Peking University, Beijing 100191, China; m School of Medical Device, Shenyang Pharmaceutical University, Benxi 117004, China; n Department of Epidemiology, Radiation Effects Research Foundation, Hiroshima 732-0815, Japan; o Korea Central Cancer Registry, National Cancer Center, Goyang 10408, Republic of Korea; p Center for Public Health and Epidemic Preparedness and Response, Department of Epidemiology and Biostatistics, School of Public Health, Peking University, Beijing 100191, China; q Cancer Surveillance Branch, International Agency for Research on Cancer, Lyon 69366, France

**Keywords:** Cancer registration, Artificial intelligence (AI), Machine learning, Deep learning, Generative AI

## Abstract

Cancer has become the second leading cause of death, the global cancer burden is rapidly increasing, and there are marked disparities between and within countries worldwide. Population-based cancer registries systematically collect data on cancer patients in defined populations, which play a crucial role in planning and assessing cancer prevention and control strategies. While the development of cancer registration has been marked by increasing standardization of definitions and methods and the electronic processing of data, the advent of artificial intelligence (AI) offers opportunities to further reduce the labor-intensive nature of registry operations, particularly where registry resources are scarce. These include enabling the processing of large datasets, extracting complex or unstructured data patterns to support cancer registration data abstraction, and facilitating data quality and control. The analysis and dissemination of registry data are also increasingly integrating AI methodologies. This paper provides a comprehensive overview of the application of AI in cancer registration. We investigate the challenges associated with integrating AI into existing cancer registry structures, with a particular emphasis on network and computational constraints, uneven resource allocation, and potential biases and limitations within AI systems. We propose a forward-looking AI-enhanced framework for cancer registration, highlighting AI’s potential to optimize efficiency in cancer registration and the use of registry data for cancer control and cancer research.

## Graphical abstract

**Figure:**
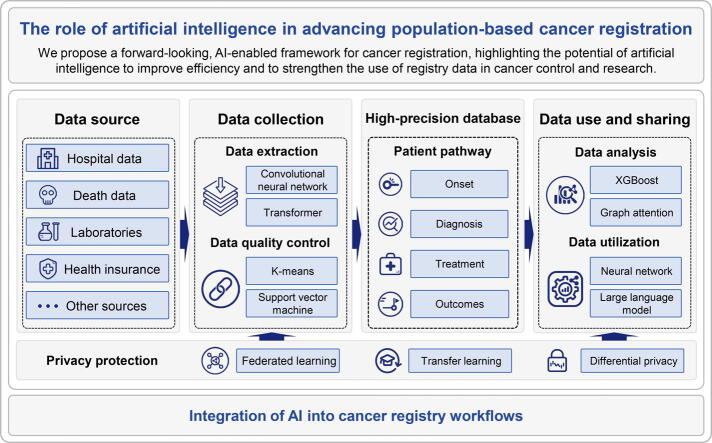

## 1 Introduction

**Fig. 1:**
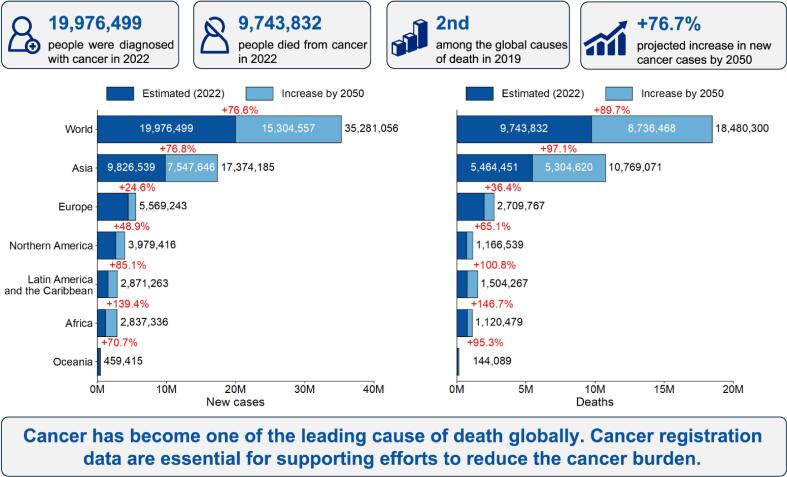
Global cancer burden in 2022 and projections up to 2050 if current rates do not change (data are from GLOBOCAN 2022).

Cancer is the second leading cause of mortality worldwide, imposing a significant burden on healthcare systems and society [1]. According to the International Agency for Research on Cancer’s Global Cancer Observatory, which derives its GLOBOCAN 2022 estimates from population-based cancer registries (PBCRs), the global number of new cases of cancer will increase from 20 million in 2022 to over 35 million by 2050, should current rates remain unchanged (Fig. 1). In particular, Asia contributes the largest current and future burden in terms of absolute numbers of new cancer cases due to its large population. On the other hand, Africa will face the largest relative increase in future cancer cases. With the global cancer incidence burden rising year-on-year, the development and implementation of effective strategies across the cancer continuum are critically needed to mitigate the burden and suffering from the disease [2].

PBCRs are systems for systematically collecting, managing, analyzing, and disseminating data on all new cancer cases that occur in a defined population from multiple sources. PBCRs have a unique role in planning and evaluating cancer control programs by facilitating the development of public health policies, resource allocation, and targeted prevention strategies [3]. Hospital-based cancer registries (HBCRs) are key sources of information for PBCRs [4]. Integrating HBCR data with electronic health records (EHRs) is increasingly common, as cancer-related information is often scattered across different departments and databases within hospitals; such linkage enables centralized access and compilation, thereby enhancing data quality.

**Fig. 2:**
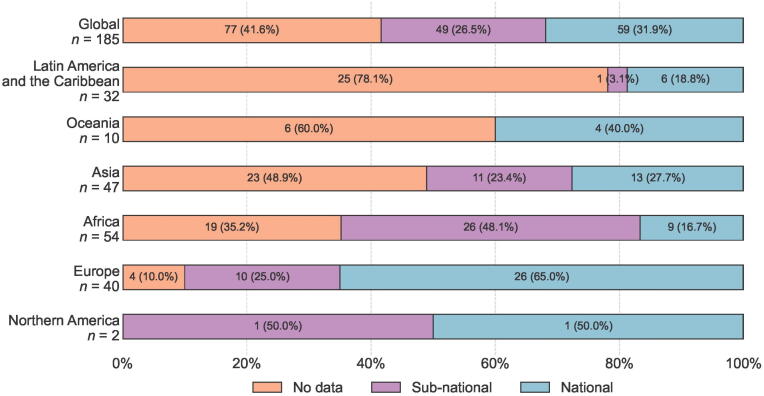
Global coverage of cancer registration data by region and type in 2022 (data are from GLOBOCAN 2022).

Historically, most PBCRs have operated through manual case identification and data registration. With technological advancements, an increasing number of PBCRs, where resources allow, are undergoing automation, whereby data are not only abstracted but also systematically coded and accurately entered into electronic systems. Despite the intrinsic value of this development, many PBCRs are still faced with multiple challenges, including a lack of governmental buy-in and a corresponding lack of resources. PBCRs are often hampered by the labor-intensive nature of data collection, as well as the potential inaccuracies in data entry and challenges in data standardization, notably in countries with restricted technical, financial, and human resources [5]. In almost 40 countries, PBCRs have yet to be established [6]. The extent of population coverage by high-quality PBCRs varies widely by world region. Transitioning countries in Africa, Latin America and the Caribbean, and the Asia-Pacific region have substantial gaps in data coverage and quality of PBCRs, requiring that national estimates are based on modelling and data sources from outside the countries (Fig. 2) [7].

Artificial Intelligence (AI) has evolved from a specialized resource into a publicly accessible tool [8], [9], [10]. With the capacity to analyze complex datasets and identify salient patterns, AI has been increasingly applied to operational tasks within the PBCRs, including the processing of clinical text and images, and the extraction of registry variables from unstructured data, with the potential to enhance data quality and increase timeliness [11], [12]. Through techniques such as natural language processing and machine learning, AI has the potential to optimize the accuracy, efficiency, and completeness of collected cancer data, leading to a more robust cancer registration framework [13], [14], [15]. However, PBCRs are facing challenges when integrating with AI technologies, including data privacy concerns, algorithmic biases, hallucinations in large language models (LLMs), and the substantial computational requirements, with the extent of AI adoption varying considerably across world regions and healthcare systems.

In this review, we introduce the core concepts of AI and its potential applications in PBCRs, both from the perspectives of data collection and data utilization. We highlight the unique context and technical challenges in the use of AI and explore innovative solutions by leveraging next-generation information technologies dedicated to PBCR. We aim to offer insights that will facilitate the effective translation of AI into PBCR practice, ultimately leading to the increased national coverage and quality of cancer data from PBCRs.

### 1.1 Search strategy and selection criteria

We conducted a comprehensive literature review on Web of Science, PubMed, Wanfang, Embase, and Scopus databases, without language or date restrictions, for articles published before Dec 19th, 2025, related to cancer registration and AI. We employed a broad set of keywords and their synonyms, including AI methodologies (for example, artificial intelligence, machine learning, deep learning, convolutional neural networks, recurrent neural networks, transformers, and large language models) and cancer registration contexts and tasks (for example, cancer, population-based cancer registries, cancer registration, registry, data extraction, information extraction, coding, and abstraction). Titles and abstracts were screened for relevance to core PBCR functions, including case ascertainment, abstraction, and coding of registry variables, data linkage, and quality control, with full texts assessed where necessary. We included studies relevant to population-based cancer registration, and we identified additional papers through citation tracking. We organized and synthesized our review according to the PBCR workflow and AI methodology categories.

## 2 Current status and specific roles of AI in cancer registration

### 2.1 Brief introduction of AI methodologies

**Fig. 3:**
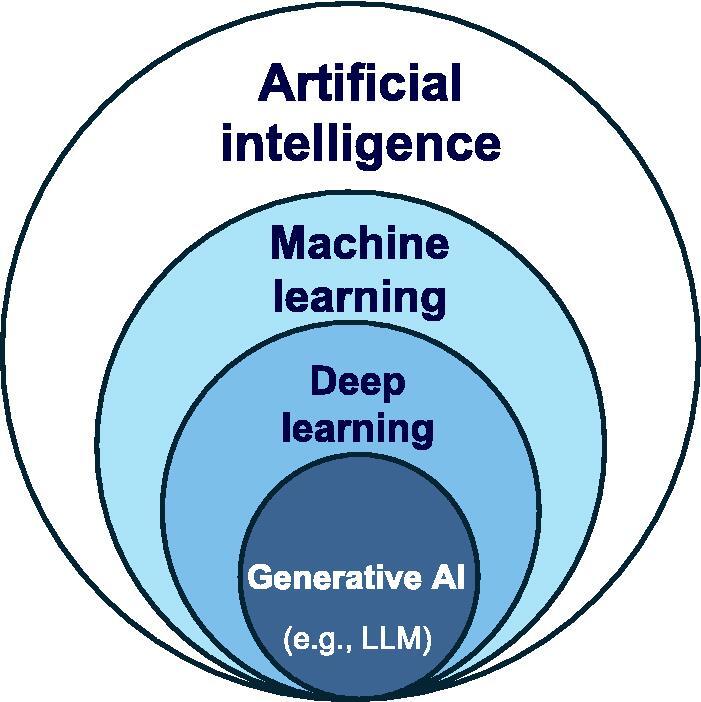
Hierarchy of AI, machine learning, deep learning, and generative AI.

There are multiple subfields of AI that can be useful for cancer registration, including machine learning, deep learning, and generative AI techniques. Fig. 3 visualizes the relations between these concepts. Machine learning typically uses engineered features or representations from raw data, with models such as support vector machines and logistic regression used for pattern recognition and prediction. Deep learning, a subfield of machine learning, employs neural networks to learn features directly from unstructured inputs such as EHR text, clinical images, and surgical videos. Generative AI, a deep learning approach, creates content by modelling data distributions. Language and vision–language generative models can produce text, images, and audio and support interactive applications. Table 1 compares these methodologies in the context of cancer registration, detailing their representative techniques, methodological features, computational requirements, and applicable scenarios.

**Table 1:** Comparison of AI methodologies for cancer registration.

AI method	Techniques	Methodological features	Applicable scenarios	Advantages of AI method
Machine learning	XGBoost, transfer learning, isolation forest, local outlier factor, support vector machine, decision tree, random forest, gradient boosting	Feature engineering, structured data, ensemble methods, and manual preprocessing	Cancer classification, mortality and survival prediction from cancer registration data	Interpretability, low computing cost, easy deployment
Deep learning	Convolutional neural network, transformer, recurrent neural network, hierarchical attention network, graph attention network, federated learning	Handling unstructured data (text, images), end-to-end learning, automatic feature extraction, and large datasets	Text/image processing, extracting registry variables from unstructured data, federated analysis without raw data sharing	Multimodal/cross-modal analysis, deep feature mining
Generative AI	Large language model, vision-language model, prompt engineering, retrieval-augmented generation	Generation and interaction, generalization and emergence	Inferring cancer registry variables from EHRs, using agents for data analysis	Reasoning ability, knowledge updating, and generalization capability

#### 2.1.1 Machine learning and deep learning

*Machine learning* is characterized by its ability to automatically learn patterns from data, thereby reducing the reliance on explicitly programmed task-specific rules [16]. However, certain predefined steps, such as selecting relevant features and determining how to handle missing values, are still required. Rule-based deterministic algorithms (e.g., string matching) enable structured coding but can miss complex or ambiguous information. Machine learning models such as Support Vector Machines (SVMs) [17] and extreme gradient boosting (XGBoost) [18] can learn patterns from clinical text using bag-of-words features. Beyond text processing, machine learning is also applied in cancer registration to detect data inconsistencies through anomaly detection. By utilizing data-driven approaches, machine learning addresses the limitations of rule-based systems, particularly in processing heterogeneous and unstructured data [19]. This capability enables the automation of data abstraction in cancer registration, reduces reliance on manual review, and decreases the risk of human error [20].

*Deep learning*, a specific branch of machine learning, uses neural networks such as convolutional neural networks (CNNs) [21] for image recognition tasks and recurrent neural networks (RNNs) [22] for capturing sequential information in text. Its core capability, automated feature extraction (also known as representation learning), removes the need for manual feature engineering [23]. These models learn hierarchical features directly from raw, unstructured data (e.g., pathology reports), enabling them to capture complex context and semantics [24]. Consequently, these models are increasingly applied to automate core tasks in cancer registration. For example, they can extract tumor characteristics from pathology reports, including primary tumor site, histologic grade, and tumor-node-metastasis (TNM) stage.

#### 2.1.2 Federated learning

*Federated learning*, a distributed approach, may address data privacy and security by allowing multiple hospitals or cancer registries to train a shared AI model collaboratively without exchanging sensitive patient information [25]. Instead of pooling data in a central repository, each participating site keeps patient-level records locally and contributes only learning updates under an agreed protocol [26]. This decentralized framework permits the development of robust models across institutions and enables multicenter collaboration while ensuring that patient data remain protected within each registry. In the context of PBCRs, this approach is relevant because data are often fragmented across regions and governed by strict regulations.

#### 2.1.3 Generative AI

*Generative AI* advances content creation through language and vision-language models [27]. This technology moves beyond pattern recognition to tasks requiring reasoning, as models are pre-trained on vast domain knowledge [28]. In cancer registries, this enables AI to analyze unstructured EHR data, such as pathology reports and discharge records, to extract and infer essential data elements, including the date of diagnosis and the International Classification of Diseases for Oncology (ICD-O) codes. Generative AI can also produce synthetic medical data that is statistically similar to real patient data [29]. This approach may provide a potential strategy to deal with the challenges of data scarcity and privacy in future applications. Together, the advances in AI-driven approaches offer opportunities to enhance data management, improve the accuracy of cancer registration, and support evidence-based decision-making in public health and clinical settings.

### 2.2 AI applications in data collection and integration

#### 2.2.1 Cancer registration data collection: evolution from traditional methods to EHR integration

PBCRs rely on multiple sources of information on cancer cases in the target population. PBCR data are collected and organized in accordance with international cancer registry standards, using coding systems such as ICD-O and TNM. As certain items are not directly available within patient records, data interpretation is required. Historically, cancer registration has primarily relied on manual processes for case identification and extraction of structured data from hospitals’ unstructured sources, such as pathology reports, medical records, and imaging results [30]. Registration staff review patient records to gather key information, including demographic details (e.g., name, age at diagnosis, and address) and tumor characteristics (e.g., anatomical site, histological type, and stage at diagnosis) [4]. These data are recorded on paper-based forms, aggregated within hospitals, and periodically submitted to regional cancer registries. Registry staff then manually entered them into digital databases [31].

Such a workflow has several limitations. It depends heavily on skilled data-entry personnel, increasing the risk of transcription errors and inconsistencies. Manual verification is labor-intensive and time-consuming, leading to inefficiencies and potential delays. Resource constraints also restrict the range of variables collected, excluding valuable details such as treatment outcomes or genetic information [32], [33], [34]. Together, these challenges limit the efficiency, accuracy, and scope of traditional cancer registration systems.

While the above operational model is still used in some countries, advances in electronic and information technologies have meant that cancer registration procedures have changed substantially in many countries. Registries increasingly adopt software systems that use electronic records to construct consolidated cancer files. An increasing number of countries have integrated cancer registration with EHRs. By leveraging structured and unstructured EHR data, these systems can automate case finding and reduce manual workload. Provisional registrations are subsequently validated through manual review to ensure data accuracy. Such semi-automated systems have demonstrated high sensitivity and validity.

#### 2.2.2 AI for information extraction

Unstructured text data in EHRs remain a critical information source for cancer registries, and deep learning has been applied in pilot studies to extract structured registry variables from such text and medical images [35], [36], [37]. Table 2 summarizes key studies on the role of AI in cancer registration. Through a multi-task learning design, CNNs can extract several reportable cancer registration variables from unstructured text without increasing training or inference time. Qiu et al. [45] applied a CNN to identify primary cancer sites in unstructured pathology reports, illustrating the value of deep learning for automated information capture. Mitchell et al. [46] applied a Transformer-based question-answering system that tolerates varied terminology, word order, and spelling while extracting tumor locations and histological descriptions from free-text pathology reports. Alawad et al. [47] used deep transfer learning with convolutional neural networks trained on pathology report text from the Louisiana and Kentucky PBCRs in the U.S. With a certain level of accuracy, their model extracted variables such as primary tumor site and morphology, demonstrating transferability across registries.

**Table 2:** Summary of key studies on the role of artificial intelligence in cancer registration.

Authors	Year	Data source	Method type	Feature	Application area	Effect, problem solved
Chen et al. [38]	2024	SEER data	Deep Learning - CNN	Capturing local patterns and hierarchical structures	SEER cancer registry data processing	Reduced reporting delays, enabling near real-time cancer incidence reporting
Yang et al. [39]	2024	Unstructured EHRs	Deep learning - Hybrid Neural Symbolic System	Multi-rule abstraction, hybrid neural-symbolic learning	Automated abstraction of 40 registry elements	Improve coding quality, reduce labor and time for data abstraction
Baghdadi et al. [40]	2019	Death certificates	Machine Learning - SVM	High-dimensional classification with generalization ability	Reactive mortality surveillance	Real-time classification of death certificate data
Röchner et al. [41]	2023	Cancer registry Rhineland-Palatinate	Deep Learning - Autoencoder	Identify rare and atypical patterns in high-dimensional categorical data	Quality control of EHRs	Reduced manual review efforts by approximately 3.5 times
Jung et al. [42]	2024	EHRs across the Danish Population	Knowledge Reasoning - Bayesian Cox	Model dynamic, time-varying patient risk factors	Cancer risk stratification	Risk prediction for expanding data utilization
Lu et al. [43]	2024	Histopathology images	Generative AI - Vision-Language	Multimodal integration for understanding image-text relationships	Extract cancer information	Automatically extracts cancer types from pathology data
Fang et al. [13]	2024	Distributed medical records	Deep Learning - Federated Learning	Decentralization and secure aggregation of participants	Multi-hospital cancer registry data analysis	Privacy-preserving collaboration in cancer research
Kumar et al. [44]	2023	EHR data from hospital-based cancer registry	Deep Learning - CNN, Federated Learning	Keep data localized and computations on encrypted data	Data use in multi hospital-based cancer registries	Privacy-preserving collaborative model training in decentralized EHRs

SEER: Surveillance, Epidemiology, and End Results Program; CNN: convolutional neural network; SVM: support vector machine; EHR: electronic health record.

Additionally, researchers developed a hospital-based AI system that automates the extraction of cancer registry data from unstructured EHRs, using an ensemble voting strategy that integrates three subsystems [39]. Dai et al. [48] developed a hybrid neural-symbolic system that integrates natural language processing, deep learning, and a rule-based expert system. This system automates lung cancer registry coding from unstructured EHRs, reducing coding errors and improving registry accuracy. Lu et al. [43] developed a vision–language foundation model that integrates medical imaging and textual data, enabling automated identification of cancer types in pathological data. By incorporating medical images into the analysis, diagnostic precision can be improved, which may in turn enable cancer registries to achieve more accurate coding.

#### 2.2.3 AI for data integration and workflow integration

AI has the potential to expand data use by supporting cross-domain linkage, integrating and harmonizing information from diverse sources [49]. Manual or simple deterministic linkage of hospital records, public health databases, and surveys is limited by inconsistent formats, coding standards, and incomplete data [50]. Consequently, many repositories remain isolated, restricting cross-sectional and longitudinal research. Röchner et al. [51] applied gradient boosting and neural networks to link large EHRs with German PBCR data, achieving higher matching accuracy than manual methods. In Japan, Ubie Inc. piloted a project with Kameda General Hospital, linking the hospital’s electronic medical record system to its generative AI platform via a data warehouse to support cancer registration [52].

In North America, the Surveillance, Epidemiology, and End Results (SEER) Program at the National Cancer Institute employed a machine-learning application programming interface to extract core variables, such as tumor site, histology, and laterality, from unstructured electronic pathology reports automatically [38]. Kalra et al. [14] combined supervised classifiers, including SVM and XGBoost, to automate cancer classification from pathology reports in Canada, and streamlined the encoding process for cancer registries. Similarly, Langhout et al. [53] validated an automated extraction system against manually curated cancer registry data. The results confirmed that machine-learning pipelines can accurately and efficiently capture core variables such as site, morphology, and stage from clinical narratives.

### 2.3 AI applications in data quality and control

Quality control, alongside data collection and integration, has long depended on continuous staff training to maintain the integrity, validity, and consistency of data at the PBCRs [30]. Registry workers follow standardized guidelines for classifying and coding new cases, and manual review remains the principal safeguard against errors. This review involves cross-checking multiple sources, such as pathology reports and medical records, to reconcile discrepancies and ensure uniform coding [54]. Although these methods provide a foundation for quality, they are labor-intensive, time-consuming, and prone to human error. As the volume and complexity of unstructured data increase, manual procedures can be overwhelmed, leading to incomplete or inconsistent records [55]. These limitations have prompted growing interest in AI-assisted approaches for PBCR data quality and control.

Validity, defined as the proportion of registry cases that truly possess a given attribute, depends on the accuracy of source documents. The precise requirements for a cancer registry depend to a large extent on the local capacity of medical diagnostic services. AI has the potential to improve data quality by detecting errors, standardizing entries, and automating validation. Tabassum et al. [56] applied unsupervised techniques (Isolation Forest, Local Outlier Factor) and supervised classifiers (SVM, decision tree, random forest) to identify inconsistent patient records in EHRs in the UK, achieving accuracy up to 99.21% while reducing false positives. Similarly, Estiri et al. [57] in the U.S. used a hybrid hierarchical k-means algorithm to identify implausible laboratory and vital sign values, attaining specificity greater than 99.97% with minimal false positives. Compared with rule-based methods, this approach is more suitable for large and diverse EHR data, where predefined thresholds are difficult to maintain and prone to misclassification. These studies indicate that AI may enhance the quality of EHR data on which cancer registries rely.

AI has the potential to streamline the detection of inconsistent coding and data entry across large-scale cancer registries, thus strengthening data uniformity and reliability. Unsupervised dimensionality-reduction methods such as autoencoders identify anomalous entries in EHRs without labelled data or manual guidance, providing faster analysis [41]. Röchner et al. [58] developed a specialized scoring system from an autoencoder to pinpoint specific implausible variables within anomalous cancer records, demonstrating its ability to identify the source of errors through validation by medical coders.

Completeness in cancer registration refers to the extent to which all cancer cases within a defined population are accurately captured by the registry. Incidence and survival proportion approach their true values only when case-finding procedures achieve maximal completeness. Case-finding is often more difficult in older adults, who receive fewer pathological diagnoses. AI-assisted interpretation of clinical data, such as medical imaging reports, can help clinicians confirm diagnoses and reduce missed cases, which may have the potential to improve the completeness of cancer registry data [15].

### 2.4 AI applications in data analysis and more comprehensive data use

Improvements in data quality and control form the basis for subsequent applications of AI in PBCR data analysis, where methods increasingly support more comprehensive and integrated use of health data. Conventional statistical methods, such as survival analysis and the Cox proportional hazards model, are commonly employed to estimate outcomes, and conventional statistical approaches are generally sufficient for routine PBCR cancer reporting. They are grounded in well-established statistical theory, provide interpretable effect estimates (e.g., hazard ratios), and allow for straightforward incorporation of covariates and adjustment for confounding. Moreover, their assumptions and limitations are transparent, which facilitates validation, reproducibility, and acceptance in clinical and epidemiological research [59]. AI may unlock new value from cancer registries, extending their utility for cancer control.

As PBCRs evolve beyond their traditional role of reporting cancer incidence to support broader functions, several studies have explored AI methods for risk and survival prediction. Varlamis et al. [60] applied data mining techniques, including naive Bayes and random forests, to refine cancer data estimates and strengthen survival prediction. Ganta et al. [61] used a random-forest model to predict 180-day cancer-mortality risk across racial subgroups based on New York data from the Mount Sinai Health System registry linked to EHRs. A multi-task deep-learning model predicted both peritoneal recurrence and disease-free survival in gastric cancer by analyzing pre-operative CT images, illustrating expanded data utilization [62].

**Fig. 4:**
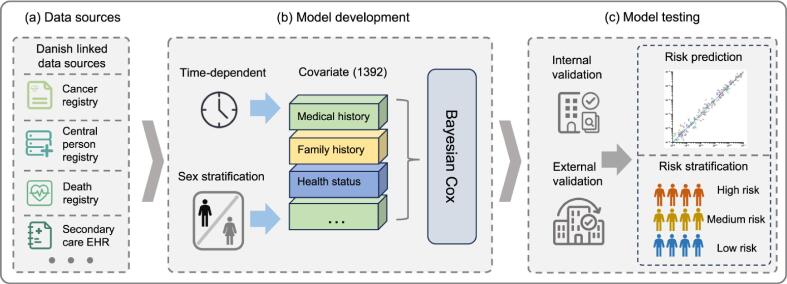
Example of a population-based registration data linking with EHR data for multi-cancer risk stratification: a retrospective modelling and validation study from Denmark [42]. (a) Danish linked population health data sources. (b) A Bayesian Cox model for multi-cancer risk estimation. (c) Internal and external validation for risk prediction and multilevel risk stratification.

Linking cancer registry records with screening and primary care data can support early detection research, thereby broadening the use of registry data. Jung et al. [42] developed a multi-cancer, population risk-stratification model using Danish PBCR data within a Bayesian Cox framework (Fig. 4) and achieved a mean C-index of 0.81 compared with 0.59 for the baseline model.

### 2.5 AI applications to maintain data confidentiality

Throughout the PBCR workflow, protecting data confidentiality remains a core requirement. The success of PBCR operations depends on the collaboration of clinicians, pathologists, and staff in administration to ensure access to data. Privacy safeguards once relied on manual review and basic encryption, leaving large-scale collaborations open to data-security breaches [63]. Data confidentiality laws vary from country to country. Given that cancer registries do not directly collect information from patients but rely on secondary sources, asking for informed consent is impractical. Privacy regulations and fragmented health-information systems can restrict inter-institutional data sharing and multicenter research in many settings, lowering collaboration rates. Privacy-preserving techniques, such as federated learning, may support multi-institutional collaboration by retaining raw data within PBCRs and hospitals while sharing only model parameters.

Federated learning shares only models, which helps protect patient-level information throughout the analytic process. Fang et al. [13] created a framework based on federated learning that allows several hospitals in the U.S. to train mortality prediction models without exchanging EHRs. Wenzel et al. [64] applied a federated approach to identify women with early-stage cervical cancer who have a low risk of lymph-node metastasis, while keeping all sensitive data on-site. The Nordcan.R tool facilitates federated analysis and quality assurance of cancer registry data by generating statistics without transferring identifiable information beyond the confines of each PBCR, thereby adhering to data protection standards [65]. This decentralized strategy mitigates the risk of privacy breaches while enhancing analytical precision and operational efficiency.

When integrating AI into PBCR operations, privacy protection should run through the workflow. Federated learning may enable cross-registry data use while keeping source data on site. Locally deployed open-source LLMs can reduce privacy risk for LLM-based abstraction [66]. Even so, workflow governance and clear role-based access control remain essential.

## 3 Challenges and limitations in the use of AI in cancer registration

### 3.1 Infrastructure and resource constraints

AI has the potential to transform PBCR workflows, but implementation still faces challenges and limitations. AI cannot be effectively deployed in regions where cancer registration infrastructure remains underdeveloped or is entirely lacking. Funding constraints significantly undermine the quality and availability of training and capacity-building initiatives. In South Africa, outdated infrastructure and insufficient fiscal support impede digital transformation strategies within public healthcare systems [67], and computer-based medical information systems remain underdeveloped. Similar problems occur in other transitioning countries, where unstable economic conditions and scarce resources may obstruct the implementation of PBCRs [68], [69]. This highlights the need for strategic investment and policy reforms to support capacity building.

In practice, the deployment of AI tools is further constrained by computational resources. The increasing scale and complexity of AI models, such as GPT-5 (https://openai.com/index/introducing-gpt-5/) or DeepSeek-R1 [70], require high-performance graphical processing units and large data storage capacity [71]. While smaller, task-specific language models can perform well with modest resources, the overall computational burden remains a significant hurdle [72].

### 3.2 Growing inequalities

The quality and availability of cancer registry data are skewed towards well-funded institutions in transitioned regions. The Nordic countries, for example, produce high-quality national statistics that support reliable model training, whereas several other countries, like Afghanistan and Madagascar, lack registry data and rely on estimates [73]. Similarly, in Ethiopia, reliance on manual data collection leads to challenges in ensuring complete datasets [68], [74].

Consequently, the deployment of AI-enabled registration systems is concentrated in technologically advanced regions. Countries with mature electronic health-record infrastructures, such as the U.S. and the U.K., may achieve real-time cancer surveillance, but nations with limited internet access and outdated data systems may struggle to implement similar tools [75], [76]. Without action, this digital divide may prevent transitioning countries from fully benefiting from the potential of AI-enhanced registration and analysis.

### 3.3 Trust and interpretability

The routine adoption of AI may be constrained by distrust in AI technologies, limited transparency of the underlying systems, and the absence of sufficient institutional incentives for healthcare institutions to embrace such tools. Moreover, AI-based methods are not always superior to traditional statistical approaches; the performance depends on the data characteristics. Trust in AI remains a critical challenge, as many systems function as black boxes, giving little insight into how inputs produce outputs [77], [78]. In addition, updates to routine models can alter outputs without clear notice, weakening reproducibility. Beyond these technical issues, users may resist unfamiliar tools because of perceived risks to safety and liability.

### 3.4 Ethical and legal concerns

Ethical concerns are also a limiting factor for AI development in cancer registration. Much of the reported evidence comes from simulations or theory-driven demonstrations, so real-world validity and safety remain uncertain [79]. Risks include inaccurate outputs, systematic bias against underrepresented groups, privacy leakage through model training or prompts, and LLM hallucinations that produce false statements [80]. Responsibility and accountability are unclear when automated suggestions influence registry actions. Embedding AI into cancer registry workflows therefore requires privacy-preserving data management, explicit role definitions and escalation paths, documented model and data provenance, ongoing impact assessment, and human oversight at multiple decision points. Legal concerns persist regarding who should be held accountable for errors arising from AI-assisted registration.

### 3.5 Validation, accountability, and data completeness

SEER has piloted an AI-supported path-centric workflow with an initial submission approximately 2 months after the end of the diagnosis year, representing near “real-time” data collection and yielding delay-adjusted incidence rates comparable to the conventional 22-month standard [38]. However, exclusive reliance on these approaches may limit opportunities for thorough validation, thereby increasing the risk of incompleteness or inaccuracies, which may ultimately compromise data quality. AI models require regular retraining to keep up with changes in medical terminology and cancer classification systems. Mechanisms for systematic validation and transparent oversight are urgently needed.

## 4 Future directions

### 4.1 Human and AI within cancer registration: human in the loop

The challenges outlined above point to future work on human-in-the-loop implementation, where registrar expertise remains essential. AI systems must be directed by domain experts to perform the intended analyses, extract relevant data, and validate outputs. Human-in-the-loop approaches are appropriate for tasks requiring nuanced judgment, such as case ascertainment and the coding of pathology reports [81]. Active learning strategies can further enhance efficiency by directing uncertain or high-value records to registrars for expert review and labeling, thereby reducing annotation demands while simultaneously improving model performance. The incorporation of explainable and interactive interfaces strengthens transparency by elucidating the rationale underlying AI-generated recommendations. Such features enable registrars to evaluate recommendations before implementation, thereby promoting user trust and the safe deployment of AI-assisted cancer registration.

### 4.2 Empowering cancer registration through the Internet of Healthcare Systems

**Fig. 5:**
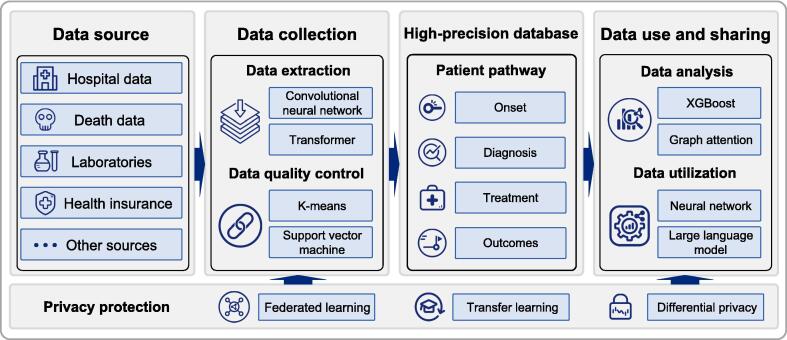
Integration of AI into cancer registry workflows.

The Internet of Healthcare Systems (IHS) is a system-level concept in which the Internet takes root in healthcare systems. It links people, objects, and information across healthcare-related entities such as hospitals, insurance, logistics providers, and research institutions [82]. Within this framework, PBCRs may move toward an AI-assisted model by expanding data sources and using multimodal technologies to improve data processing and data use [83], [84]. Additionally, AI-generated content technologies, such as agent-based workflows, have the potential to support policy formulation and equity in cancer care (Fig. 5) [85], [86]. Future developments can be conceptualized around three core innovations: (1) novel and additional data acquisition, (2) advanced technologies for data analysis, and (3) intelligent decision support systems. These innovations aim to enhance data integration, enable effective human-machine collaboration, and support precise cancer management.

The engineering-oriented development paradigm of the IHS has also been applied to the strategic development of PBCRs in China. In 2019, a unified national digital platform for PBCR data management and quality assurance was established, encompassing data entry, management, quality control, and training [31]. The national cancer registration workflow has been progressively modernized, transitioning from predominantly manual abstraction to semi-automated processes. By 2025, China operates 2806 PBCRs, covering more than 98% of the national population. Within the concept of enabling integrated use of PBCR data, programs of cancer screening, and cancer treatment data from multiple hospitals, the National Cancer Center is continuously optimizing the National Cancer Prevention and Control Platform, which may maximize the value of cancer surveillance information. This platform is currently used by more than 6000 institutions across all provinces in Chinese mainland [87].

Recent advances in open-source LLMs, such as DeepSeek, together with the availability of multi-level electronic medical records, offer new opportunities to further refine cancer registration processes [88], [89], [90]. In a Chinese pilot initiative, we applied an LLM-based system to EHRs to automatically extract key cancer registration variables [91]. The LLM-based workflow demonstrated ICD-O-3 coding performance comparable to, and in some tasks exceeding, that of expert registrars, while substantially reducing manual abstraction effort. The development of China’s PBCRs suggests a demand-led, innovation-driven model in which registration capacity evolves to meet prevention goals and registry data support cancer control.

### 4.3 Multimodal data analysis and decision-making intelligence

Multimodal analysis has the potential to extend PBCR data use by linking registry records with imaging. Imaging may help identify pathological subtypes and add clinically relevant detail beyond coded fields. Multi-source fusion and cross-institutional analytics could improve interoperability, support precision cancer surveillance, and personalized patient management. Multimodal fusion enhances data collection and utilization of cancer registration data. For example, AI for medical imaging can enable the preliminary classification of likely cancers and, combined with other sources, may reduce the time to diagnostic confirmation. Incorporating diverse modalities further facilitates more comprehensive analyses and strengthens the overall depth and quality of cancer research [92]. Multimodal data integration may also facilitate the creation of intelligent data governance platforms [93]. These platforms may automatically flag data inconsistencies and errors for human review and assist with corrections, improving data collection and storage processes [94].

Foundation models based on deep learning have advanced medical AI by using large datasets, high computational power, and advanced architectures. LLMs such as GPT for clinical text and vision models trained on medical imaging show capabilities in medical report generation and abnormality detection, supporting diagnostic processes [74], [95], [96]. AI agent technology [97] is being integrated into medical decision support systems, including simulated environments like “Agent Hospital”, where agents represent doctors, nurses, and patients [98]. However, these systems are largely experimental and must be used with human oversight.

Decision intelligence may broaden PBCR outputs beyond reporting toward decision support for patients and policy. Decision intelligence has the potential to strengthen cancer registration at both the individual and system levels. For individuals, it may support physicians through human-computer interaction and retrieval-augmented generation [99]. These methods use past diagnostic data for case comparison, supporting rather than replacing clinical decision-making [100]. For organizations, it could support PBCR planning and evaluation, such as evaluating policy impacts on cancer trends [101]. Policy interpretation agents can analyze large datasets of policy documents and implementation outcomes to relate policies to effects [102]. With feedback mechanisms, such as Agentic Workflows, these agents can support policy formulation and implementation.

## 5 Conclusion

AI is improving PBCR in certain settings, from data collection to data use. It offers opportunities for workflow automation, improving accuracy, expanding data scope, and strengthening data utilization. However, the integration of AI with cancer registration still faces challenges such as network and computing power limitations, as well as data security issues.

Looking forward, the safe and sustainable integration of AI into routine PBCR workflows will depend on secure digital infrastructure, robust governance, and transparent regulatory frameworks. Emerging approaches, including federated learning, integration of LLMs, and privacy-preserving data-sharing architectures, offer promising pathways to harness AI while safeguarding data confidentiality and public trust. Ongoing efforts to strengthen and modernize cancer registration systems will be central to future public health policymaking and cancer control, ensuring that PBCRs continue to evolve as reliable, high-quality resources in an increasingly data-driven landscape.

## Conflict of interest

The authors declare that they have no conflict of interest.
